# Cerebral Hæmorrhage.—III
*A Lecture delivered at the London School of Clinical Medicine.


**Published:** 1912-04-27

**Authors:** Guthrie Rankin

**Affiliations:** Physician to the "Dreadnought" and Royal Waterloo Hospitals.


### CEREBRAL HÆMORRHAGE.—III.*

By GUTHRIE RANKIN, M.D. Glas., F.R.C.P. Lond. and Edin., Physician to the "Dreadnought" and Royal Waterloo Hospitals.

Epilepsy: The coma which ensues upon an epileptic seizure may be mistaken for apoplexy, but there is generally a record of previous similar attacks to guide the diagnosis, or it may be ascertainable that the present comatose state was preceded by a convulsive seizure. The face, though at first cyanotic, soon becomes pale, and the respiration rapidly changes from its early stertorous character to a distressing moaning cadence. The pupils are widely dilated and irresponsive to light, and there is complete muscular relaxation. After about ten or fifteen minutes there is a gradual return to consciousness, and the patient falls into a natural sleep. It is often found that, during the attack, he has bitten his tongue or passed water involuntarily. There is no subsequent paralysis. The commencement of the epileptic seizure is always sudden, whereas the onset of the apoplectic attack is most frequently gradual.

Opium Poisoning: The coma produced by an excessive quantity of opium is of gradual onset, but where the dose has been sufficiently large it is ultimately very deep. The pupils are closely contracted; the respirations are slow and full; the face is suffused and often cyanosed; the temperature is normal or slightly lowered; the skin is warm and moist; and the flavour of the drug may be detected in the breath or its presence demonstrated in the stomach contents. There is an entire absence of convulsion, pyrexia, or localised paralysis. In pontine cases, where the pupils resemble those met with in opium poisoning, there is often evidence of a crossed paralysis, and the temperature, though low at first, becomes, within a few hours, febrile.

Diabetes: Coma frequently supervenes suddenly in diabetic cases, but the history of the illness, the youth of the patient, the emaciation, the peculiar, sweet, chloroform-like smell of the breath, and the presence of sugar and acetone in the urine combine to form a symptom-complex which is unmistakable.

The treatment of cerebral hæmorrhage must be regarded from two points of view—curative and preventive.

Curative: When a sufficient cause can be found to account for the apoplexy, treatment must be directed to its removal or relief, but so many cases occur where it is impossible to say definitely whether hæmorrhage or thrombosis is the exciting factor that general principles only can be laid down; a considerable margin must be left for filling in such details of management as the circumstances of each case suggest. Absolute rest in bed is essential, and the less the patient is moved about after the occurrence of his "stroke" the better. He should lie with his head and shoulders well raised, and he is best placed on his side to lessen the stertor of his breathing by preventing his tongue from falling back on his palate. All articles of clothing that constrict the neck must be loosened. Sinapisms over the chest and abdomen or to the calves of his legs serve to restore warmth and may have some effect in reducing the volume of blood in the brain. An ice-bag should be applied to the head. The bowels ought to be freely opened, and this is best accomplished by five grains of calomel or two minims of croton oil put upon the patient's tongue. Where there is evidence of pronounced cerebral congestion and excessive vascular tension, venesection should be resorted to without hesitation, it being, however, borne in mind that this procedure is only justified when hæmorrhage is the probable lesion responsible for the condition. If bleeding from the arm, which is the best and speediest method of relieving the strain on the vessels, is objected to, six or eight leeches may be applied to the temples. Lumbar puncture with a view to relieving the compression by lowering the blood pressure is sometimes justifiable. When there is difficulty in swallowing, the risk of food getting into the air-passages and setting up pneumonia should be avoided by withholding for the time being the administration of food and drink by the mouth. The patient can be kept going comfortably over several days by rectal alimentation. As soon as possible, diuretics ought to be administered, and they ought to be combined with bromides if muscular twitchings indicate the possibility of the occurrence of convulsions. The ordinary styptics, such as ergot, hamamelis, adrenalin, gallic acid, etc., are of little value, but nitro-glycerine and the nitrites are, in all cases where the tension is high, of considerable use. In acute meningeal cases, trephining, removal of the clot, and ligature of the ruptured artery are the obvious indications.

When consciousness returns, every kind of noise and excitement should be guarded against. The bowels ought to be freely opened every day. Diuretics ought to be continued and aconite or antimony should be added if the febrile symptoms, during the reactionary stage, are at all pronounced. A succession of small fly-blisters to the nape of the neck may also be useful in assisting the relief of the drowsiness. The diet should continue simple and sloppy, but may be added to gradually as the power of mastication and swallowing improves. Later on, massage, passive movements, and the application of the Faradic current to the paralysed limbs will promote their recovery and minimise the risk of permanent contractures. Nervine tonics, such as quinine, strychnine, phosphorus and arsenic will promote the restoration of those nerve elements which are suffering only from slight damage or from pressure effects. Iron, the iodides, and cod-liver oil, together with change of scene and cheerful surroundings, are promotive of convalescence when the right time comes for recourse to them. Special care must be taken, by the use of a water-bed, frequent change of position, and the application of spirit lotions, to provide against the occurrence of bed-sores. The bladder must be watched, and emptied periodically if necessary.

Preventive: When a person of advanced life

* A Lecture delivered at the London School of Clinical Medicine.

complains of headache, giddiness, and other suggestive premonitory symptoms especially if such a person has had an apoplectic seizure, a threatened attack may be warded off by keeping him quietly in bed, applying ice to his head, administering a brisk purge, and reducing arterial tension by the use of iodides combined with nitro-glycerine.

In extreme cases even blood-letting may be justifiable as a prophylactic measure. A person who has once suffered from apoplexy should, throughout the remainder of his life, avoid excitement and hurry, live plainly, take little or no stimulant, spend ten or twelve hours out of the twenty-four in bed, carefully regulate his bowels, and look with suspicion on any headache or vertigo which he may experience.

Ligature of the common carotid artery has been suggested as a useful measure against the recurrence of intracranial hæmorrhage in cases where it has once happened.

